# A JOURNEY FROM GERIATRIC MEDICINE TO GEROSCIENCE

**DOI:** 10.1093/geroni/igac059.1249

**Published:** 2022-12-20

**Authors:** Luigi Ferrucci

**Affiliations:** National Institute on Aging, Baltimore, Maryland, United States

## Abstract

In 1984 LZ Rubenstein group demonstrated that geriatric assessment improved function and QoL in frail in frail, older patients. I heartly joined the international sparkle of enthusiasm generated by these results although later work did not match our expectations. Understanding the complexity of older person is an extraordinary tool for geriatricians, but coding the nuances of making the “best choice” in a randomized trial remains difficult. Frailty is difficult to reverse because it occurs when resilience is exhausted. Geroscience postulates that chronic diseases and frailty stem from the biological mechanism of aging and that interventions that slow down aging will successfully improve resilience. This approach have shown great potential but whether it will lead to prevention or improvement of frailty is unknown. While we continue to provide optimal care to frail older patients, we need to push forward the translation arm of geroscience both in area of prevention and care of older patients.

